# Factors Associated With The Covid-19 Infection Severity In Patients With Mental Disorders

**DOI:** 10.1192/j.eurpsy.2022.1304

**Published:** 2022-09-01

**Authors:** M. Turki, A. Daoud, S. Blanji, S. Ellouze, R. Ben Jmeaa, F. Ben Abdallah, N. Halouani, J. Aloulou

**Affiliations:** Hedi Chaker University Hospital, Psychiatry “b” Department, Sfax, Tunisia

**Keywords:** COVID19, psychiatry, Mental Disorders

## Abstract

**Introduction:**

Recent research showed that persons with mental disorders may represent a population at increased risk for coronavirus disease (COVID-19) infection with more adverse outcomes.

**Objectives:**

We aimed to analyze clinical profile of psychiatric inpatients during their infection with COVID-19, and to explore factors associated with the disease progression.

**Methods:**

We analyzed retrospectively the medical records of 32 psychiatric inpatients, hospitalized in psychiatry “B” department at Hedi Chaker hospital (Sfax, Tunisia), and who contracted the COVID-19 infection. We used “Charlson Comorbidity Index Score” (CCIS), predicting 10-year survival in patients with multiple comorbidities.

**Results:**

Somatic history was reported in 50% of patients. The CCIS ranged between 0 and 4. Psychiatric diagnosis was schizophrenia in 81.3% and bipolar disorder in 18.7% of cases. The clinical symptoms reported were fever (50%), dry cough (75%); dyspnea (34.4%). Biological assessment showed a lymphopenia in 40.6% and a high C-Reactive Protein (CRP) in 53.1%. Among our patients, 37,5% needed oxygen, and 25% were transferred to the intensive care unit. The COVID-19 complications were mostly bacterial pulmonary superinfections (21.9%) and pulmonary embolism (9.4%). Only three (9.4%) patients died from the virus. Patients with medical history were more likely to need oxygen (p<0.001). Clinical and paraclinical parameters associated with oxygen need were: fever (p<0.001); dyspnea (p<0.001); lymphopenia (p<0.001); high CRP (p=0.001). Patients presenting pulmonary superinfection or embolism were more likely to require oxygen (p=0.006 and p=0.044 respectively).

**Conclusions:**

This study highlighted factors that may worsen the COVID-19 infection evolution, and which require special attention, in order to improve the prognosis of this disease.

**Disclosure:**

No significant relationships.

